# A Scoping Review of Barriers to Osteoporosis Treatment and Fragility Fracture Prevention in Adults Aged 50 and Above in the United Kingdom

**DOI:** 10.7759/cureus.101511

**Published:** 2026-01-14

**Authors:** Imobhio Gregory Okhifun

**Affiliations:** 1 Public Health, Brunel University of London, London, GBR; 2 Trauma and Orthopaedics, Barking, Havering, and Redbridge University NHS Trust, London, GBR

**Keywords:** falls, falls prevention, fragility fractures, hip fractures, osteoporosis, osteoporotic fractures

## Abstract

Osteoporosis is a major public health condition characterised by reduced bone mass and impaired bone microarchitecture, resulting in an increased risk of fragility fractures. In the United Kingdom (UK), osteoporotic fractures-particularly of the hip, spine, and wrist-are associated with significant morbidity, mortality, and escalating healthcare costs. Despite the availability of effective pharmacological therapies and national clinical guidelines, a substantial treatment and prevention gap persists among adults aged 50 years and older.

The primary aim of this scoping review was to identify and synthesise the key barriers to optimal osteoporosis treatment and fragility fracture prevention in the UK. A secondary aim was to examine how individual, socioeconomic, and health system factors contribute to underdiagnosis, undertreatment, and poor adherence to osteoporosis care. A systematic search of major electronic databases, including PubMed, SCOPUS, Embase, and CINAHL, was conducted to identify UK-based studies published within the last ten years that addressed osteoporosis management, fracture prevention, service delivery, or patient engagement. Data were analysed using thematic synthesis.

The review identified multiple interacting barriers across the care pathway. Individual-level barriers included poor medication adherence, low risk perception, missed diagnostic appointments, and socioeconomic disadvantage. At the organisational level, challenges included delayed diagnosis, inconsistent initiation of therapy following fragility fractures, limited access to bone density assessment, fragmented care pathways, and suboptimal implementation of falls-prevention strategies.

This review highlights the need for coordinated, system-wide interventions to improve osteoporosis outcomes in the UK. Strategies should focus on strengthening fracture liaison services, improving access to diagnostics, supporting long-term treatment adherence, and targeting high-risk and underserved populations to reduce preventable fragility fractures.

## Introduction and background

As people age, bone mass progressively declines as bone resorption exceeds bone formation. This process typically begins between the ages of 30 and 40, and by the age of 80, the average individual has lost more than 50% of their total bone mass [1]. Excessive bone resorption leads to reduced bone strength, increased porosity, and structural fragility, a condition known as osteoporosis. Consequently, affected individuals are at a significantly higher risk of fractures resulting from minimal trauma or low-energy falls, often occurring from standing height [2].

Osteoporosis predominantly affects postmenopausal women and men aged over 50 years, with women experiencing a fivefold higher risk of fragility fractures compared to men [3]. Beyond biological factors, social determinants of health-including low socioeconomic status, limited educational attainment, and geographic location-further increase disease burden in specific populations [4]. Fragility fractures are associated with substantial morbidity, including chronic pain, physical deformity, loss of independence, and increased mortality.

The most commonly affected skeletal sites include the hip, wrist, humerus, and spine, with hip and vertebral fractures being the most disabling [5,6]. Data from the National Hip Fracture Database indicate that approximately one-third of patients who sustain a hip fracture die within one year of injury [7], and up to half of previously independent older adults fail to regain their pre-fracture level of independence [8]. Vertebral fractures, the most prevalent osteoporotic fracture type, are often caused by low-impact activities such as bending rather than falls and are associated with chronic disability in approximately 40% of patients [6]. In the UK, survival following vertebral fractures declines from 86.5% at 12 months to 56.5% at five years [9]. Mortality following osteoporotic fractures arises not only from the injury itself but also from the exacerbation of pre-existing comorbidities.

Fragility fractures, therefore, represent a primary public health concern with significant human and economic consequences [6]. In the UK, over 300,000 individuals are admitted annually with fragility fractures, incurring combined healthcare and social care costs of approximately £4 billion per year [9]. These costs are projected to increase to £5.5 billion annually by 2025 [3].

According to the Royal Osteoporosis Society, half of women and one-fifth of men aged 50 years will sustain an osteoporotic fracture during their lifetime [10]. With an ageing population, the prevalence of osteoporosis and fragility fractures is expected to rise further. Despite the availability of effective treatments and national clinical guidelines, a substantial treatment gap persists, leaving many individuals undiagnosed and untreated and resulting in preventable fractures and avoidable mortality [11]. The National Osteoporosis Guidelines Group (NOGG) and the Scottish Intercollegiate Guidelines Network (SIGN) provide comprehensive recommendations for osteoporosis management in the UK, including case finding, fracture risk assessment, and evidence-based pharmacological and non-pharmacological interventions across primary and secondary care settings [12,13].

However, despite these established guidelines, the prevalence of osteoporosis and fragility fractures among older adults remains high and continues to increase, highlighting persistent shortcomings in treatment implementation and fracture prevention [14]. To better understand these challenges, this scoping review employed the Population, Concept, and Context (PCC) framework to formulate the research question: 'What barriers to osteoporotic treatment prevent fragility fractures among at-risk older adults?' [15].

Accordingly, the objectives of this review were to examine existing evidence to identify the causes of the osteoporosis treatment gap, explore barriers to osteoporosis management and fragility fracture prevention among adults aged 50 years and older, identify gaps in the current literature, and propose evidence-informed recommendations for healthcare professionals, public health organisations, and policymakers to improve the identification and management of individuals at risk of fragility fractures.

Literature review

The primary impact of osteoporosis is the risk of fragility fractures. In the UK, about 536,000 new fragility fractures occur each year, most of which are hip fractures (79,000), spine fractures (66,000), and wrist fractures (69,000). About 322,000 other fractures affect other bones in the body [16]. Every year, the cost of treating hip fractures amounts to £3.2 billion, while osteoporotic vertebral fractures and wrist fractures account for £1.1 billion and £84 million per annum [3]. There is evidence that optimal treatment of osteoporosis can reduce the risk of fragility fractures by up to 50% [6], reducing the burden of osteoporotic fractures. Therefore, there is a need to assess current treatment and prevention strategies to identify gaps and areas of improvement.

The NOGG and the Scottish Intercollegiate Guidelines Network (SIGN) [12,13] recommend an initial fracture risk assessment in any postmenopausal woman or man aged 50 years or older with clinical risk factors for fragility fractures. NOGG adopts the Fracture Risk Assessment Tool (FRAX) used to assess the 10-year probability of fracture in a patient, while SIGN recommends QFracture (which evaluates the 10-year risk of osteoporotic fractures in the UK population). Both tools provide the 10-year fracture probability and stratify patients into four risk groups: very high, high, intermediate, and low. Patients with a very high or high risk of fragility fractures are immediately referred for a dual-energy X-ray absorptiometry (DXA) scan to measure bone mineral density (BMD), which is used to diagnose osteoporosis [17]. Patients identified as having low BMD as confirmed by DXA scan are the referred to specialist osteoporotic centers for appropriate drug therapy and regular review, while patients with low fracture risks and/or osteopenia as confirmed by DXA scan are counselled on only healthy lifestyle changes, including smoke cessation, increased physical activity, intake of calcium and vitamin D supplements, falls prevention, and exercise therapy.

These recommendations serve as a valuable guide for clinicians and health professionals to identify and appropriately manage patients at risk of osteoporotic fractures; however, compliance with these guidelines and, in turn, early identification of at-risk individuals and prevention of these fractures, has been suboptimal in the UK [11].

A 2008 national survey assessing secondary prevention of fragility fractures found that fewer than 20% of 750 orthopaedic consultants surveyed referred their patients with fragility fractures for appropriate osteoporotic management [18]. Similarly, data from the National Hip Fracture Database showed that most patients (88.3%) were not receiving any osteoporotic treatment at presentation with hip fractures, despite being at high risk for fragility fractures [19].

One prospective study conducted in Brighton and Sussex University Hospital Trusts [20] revealed that of 98 patients admitted with hip fractures within the three-month study period, about 40% (39 patients) had at least one high clinical risk factor (Diseases with bone loss, prolonged use of steroids, at least one previous fragility fracture, and low reproductive hormone levels). Only seven of the 39 patients (18%) had received osteoporotic treatment before the fracture, while only five of the remaining 59 patients (8%) had received osteoporotic therapy before the fall. Overall, more than two-thirds of the patients had a clinical risk factor for fragility fractures. More recent studies have shown a similar pattern of suboptimal compliance with guidelines. The Newcastle-85 study, a cross-sectional analysis of 739 individuals aged 85 years in Newcastle in 2006, found that of 249 individuals identified as requiring osteoporotic treatment after fracture risk assessment, only 28.6% were on osteoporotic treatment [21].

These data clearly show a treatment gap in osteoporosis management in the UK, precluding effective primary and secondary prevention of fragility fractures. This scoping review, therefore, aims to understand the barriers to effective osteoporosis management despite clear guidelines and the availability of effective treatments. This will help guide the development of new strategies and policies to improve the diagnosis and treatment of osteoporosis and, in turn, reduce the burden of fragility fractures.

## Review

Methodology

What is the current state of research on barriers to optimal osteoporotic treatment and prevention of fragility fractures in the UK? While several studies have shown that many high-risk patients are not receiving osteoporosis treatment, a scoping review is needed to identify the various barriers and challenges that drive this treatment gap. This review is essential for mapping out the existing evidence and better understanding the barriers to osteoporotic treatment and fragility fracture prevention in high-risk patients. Therefore, this scoping review aimed to explore knowledge gaps in population-based prevention of fragility fractures and to identify areas requiring further research to inform new policies and procedures that address these barriers. To ensure transparency, the scoping review adhered to the Preferred Reporting Items for Systematic Reviews and Meta-Analyses extension for Scoping Reviews (PRISMA-ScR) [22]. Table 1 shows the PRISMA-ScR checklist. 

**Table 1:** Preferred Reporting Items for Systematic Reviews and Meta-analyese Extension for Scoping Reviews (PRISMA-SCR) Checklist [22]

Section	Item	PRISMA-ScR Checklist
Title	1	Identify the report as a scoping review.
Structured summary	2	Provide a structured summary that includes (as applicable): background, objectives, eligibility criteria, sources of evidence, charting methods, results, and conclusions that relate to the review questions and objectives.
Rationale	3	Describe the rationale for the review in the context of what is already known. Explain why the review questions/objectives lend themselves to a scoping review approach.
Objectives	4	Provide an explicit statement of the questions and objectives being addressed with reference to their key elements (e.g., population or participants, concepts, and context) or other relevant key elements used to conceptualize the review questions and/or objectives.
Protocol and registration	5	Indicate whether a review protocol exists; state if and where it can be accessed (e.g., a Web address); and if available, provide registration information, including the registration number.
Eligibility criteria	6	Specify characteristics of the sources of evidence used as eligibility criteria (e.g., years considered, language, and publication status), and provide a rationale.
Information sources	7	Describe all information sources in the search (e.g., databases with dates of coverage and contact with authors to identify additional sources), as well as the date the most recent search was executed.
Search	8	Present the full electronic search strategy for at least 1 database, including any limits used, such that it could be repeated.
Selection of sources of evidence	9	State the process for selecting sources of evidence (i.e., screening and eligibility) included in the scoping review.
Data charting process	10	Describe the methods of charting data from the included sources of evidence (e.g., calibrated forms or forms that have been tested by the team before their use, and whether data charting was done independently or in duplicate) and any processes for obtaining and confirming data from investigators.
Data items	11	List and define all variables for which data were sought and any assumptions and simplifications made.
Critical appraisal of individual sources of evidence	12	If done, provide a rationale for conducting a critical appraisal of included sources of evidence; describe the methods used and how this information was used in any data synthesis (if appropriate).
Synthesis of results	13	Describe the methods of handling and summarizing the data that were charted.
Selection of sources of evidence	14	Give numbers of sources of evidence screened, assessed for eligibility, and included in the review, with reasons for exclusions at each stage, ideally using a flow diagram.
Characteristics of sources of evidence	15	For each source of evidence, present characteristics for which data were charted and provide the citations.
Critical appraisal of evidence	16	If done, present data on critical appraisal of included sources of evidence (see item 12).
Results of each source of evidence	17	For each included source of evidence, present the relevant data that were charted that relate to the review questions and objectives.
Synthesis of results	18	Summarize and/or present the charting results as they relate to the review questions and objectives.
Summary of evidence	19	Summarize the main results (including an overview of concepts, themes, and types of evidence available), link to the review questions and objectives, and consider the relevance to key groups.
Limitations	20	Discuss the limitations of the scoping review process.
Conclusions	21	Provide a general interpretation of the results with respect to the review questions and objectives, as well as potential implications and/or next steps.
Funding	22	Describe sources of funding for the included sources of evidence, as well as sources of funding for the scoping review. Describe the role of the funders of the scoping review.

Eligibility Criteria

The scoping review included only studies that met the following inclusion criteria, including studies targeting age groups at risk of osteoporosis/fragility fractures - 50+ [2]; UK-focused studies or international studies with relevance to the UK; studies that address barriers to fragility fracture prevention and osteoporosis treatment in the above population, and studies dated within the last 10 years (2014 - 2024).

All study types were included in the search, including prospective studies, randomised controlled trials, observational studies, systematic reviews, and qualitative research. Articles were excluded based on the following criteria: articles not published initially in English, articles that do not address barriers to osteoporosis treatment or/and fragility fracture risk reduction (our research focus); non-UK studies, and articles focusing on osteoporosis in animals or the human population outside the target age population.

Information Sources

**Figure 1:**
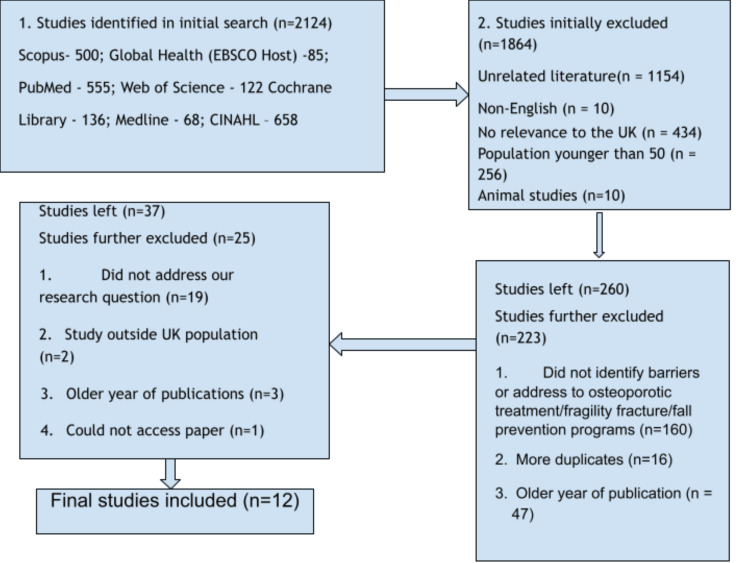
PRISMA flowchart summarizing search process [15]

Figure 1 explains the data selection process. Of 2,124 initial papers identified using the search strategy, 1,884 were excluded, primarily for irrelevance to the research focus. Of the 260 remaining articles, a further 223 were excluded, primarily for irrelevance to the research question. About 37 articles reach the final stage of screening, with 19 excluded for lack of direct relevance to the research focus. 

An extensive literature search was conducted on seven databases - Scopus, Global Health, PubMed, Web of Science, Cochrane Library, Medline, and CINAHL - related to the research question and study objectives. As shown in Table 2, the search strategy employed the PCC framework [15], utilising relevant filters from each category, including barriers or challenges, non-compliance or non-adherence. AND (osteoporotic treatment OR fragility fracture risk reduction OR osteoporotic fracture risk reduction OR fall prevention) AND (elderly population OR postmenopausal women OR older than 50) AND (UK OR Britain OR Wales OR Northern Ireland OR England OR Scotland). 

**Table 2:** Population, concept, and context (PCC) framework for search strategy [15]

PCC Component	Keywords	Terms and Synonyms used	Search string
Population	Patients aged 50 years and older in the UK	Elderly population/at-risk population	People aged 50years and older OR elderly population OR at-risk population OR United Kingdom OR Britain OR England OR Northern Ireland OR Scotland OR Wales
Concept	Barriers to osteoporotic treatment/management	Challenges/problems /non- adherence/non-compliance Osteoporosis treatment/falls prevention	Barriers OR Challenges OR non-compliance OR non-adherence Osteoporotic management OR Osteoporosis treatment OR Osteoporosis OR Osteoporosis medications OR Falls prevention OR
Context	Preventing fragility fractures	Fragility fractures/osteoporotic fractures	Fragility fractures OR osteoporotic fractures

Search

The search strategy adopted the PCC framework and was executed by the researcher [15]. Filters were applied using keywords generated in the search strategy to expand the search. The studies generated were screened using the inclusion/exclusion criteria to yield the appropriate studies (Table 3).

**Table 3:** Barriers to osteoporotic treatment and prevention of fragility fractures – search strategy [15]

Database	Search terms	Filters used	Number of articles retrieved	Reasons for exclusion	Number of articles finally included after exclusion
Scopus	Challenges OR barriers AND Osteoporotic treatment OR fragility fracture risk reduction OR falls risk reduction AND UK OR Britain OR England OR Scotland OR Wales OR Northern Ireland	None	500	Relevance to the research question, conducted in the UK Study in the last 10 years (2014-2024), targeting the population (men and women) aged 50 years and over	0
Global Health	Same	None	85	Relevance to the research question, conducted in the UK Study in the last 10 years (2014-2024), targeting the population (men and women) aged 50 years and over	0
PubMed	Same	None	555	Relevance to the research question, conducted in the UK Study in the last 10 years (2014-2024), targeting the population (men and women) aged 50 years and over	9
Web of Science	Same	None	122	Relevance to the research question, conducted in the UK Study in the last 10 years (2014-2024), targeting the population (men and women) aged 50 years and over	1
Cochrane Library	Same	None	136	Relevance to the research question, conducted in the UK Study in the last 10 years (2014-2024), targeting the population (men and women) aged 50 years and over	1
Medline	Same	None	68	Relevance to the research question, conducted in the UK Study in the last 10 years (2014-2024), targeting the population (men and women) aged 50 years and over	0
CINAHL	Same	None	658	Relevance to the research question, conducted in the UK Study in the last 10 years (2014-2024), targeting the population (men and women) aged 50 years and over.	1

Data Charting

The study’s data charting process followed the JBI methodological guidance [23] and was performed independently by the researcher. Table 4 shows the data chart table. The items included in the chart were author, year of publication, study design, sample size, setting, study objectives, study results, and study limitations.

**Table 4:** Data charting table [23]

Database	Author (Year)	Study Design / Duration	Sample Size / Setting	Study Objective	Key Findings / Results	Study Limitations
Cochrane Library	Parsons et al. [24]	Randomized controlled trial (60 months)	Multicentre primary care–based study in women aged 70–85	To evaluate the effect of population-based screening on osteoporosis treatment adherence	Low treatment initiation rates; adherence is lowest among non-screened groups	Self-reported medication use; possible recall bias
PubMed	Cehic et al. [25]	Prospective cohort study	Multicenter study involving 5456 patients aged 60 years and over across NHS hospitals in England and Wales	To assess the prescription of and adherence to medications for secondary fracture prevention following hip fractures	Poor adherence to osteoporotic medications	
PubMed	Sahota et al. [26]	Prospective observational study	200 patients attending bone density assessment	To assess attendance and adherence patterns for bone densitometry	Poor attendance for diagnostic screening and follow-up	Non-random sampling; recall bias possible
PubMed	Narayanasamy et al. [27]	Qualitative interviews	78 participants across the UK	To explore adherence to bisphosphonate therapy	Poor adherence linked to oral formulation complexity and lack of follow-up	Limited to patient-reported experiences
PubMed	Johnson et al. [28]	Retrospective cohort study	Patients in low-income regions in the UK	To examine socioeconomic disparities in osteoporosis management	Higher fragility fracture incidence and lower access to care in deprived areas	Data limited to one region; missing socioeconomic covariates
CINAHL	Gerasimavicuite et al. [29]	Descriptive cohort study (Jan 2012–2018)	8,366 care home residents aged >60 years in the UK	To describe osteoporosis-related characteristics and treatment duration among care home residents	Delayed treatment initiation and underdiagnosis; poor adherence to osteoporosis medication	Only treatments prescribed in primary care were included; vertebral fractures only
PubMed	Bennett et al. [30]	Qualitative study	27 participants aged 45–80 years across England	To assess barriers to early diagnosis of vertebral fractures	Lack of patient awareness, delays in diagnosis, and poor care coordination	Data collection overlapped with COVID-19; potential bias
PubMed	Drew et al. [31]	Qualitative interviews	20 clinicians across two hospital trusts	To explore organizational barriers to secondary fracture prevention	Poor referral and lack of protocol for post-fracture care	Findings based on limited clinical settings
PubMed	Patel R et al. [32]	Retrospective cohort study involving 178,757 patients aged 60 years and older who sustained a hip fracture in England and Wales between 1 April 2016 and 31 March 2019	Retrospective cohort study involving 178,757 patients aged 60 years and older who sustained a hip fracture in England and Wales between 1 April 2016 and 31 March 2019	To determine hospital- level organizational factors	Delay in diagnosis/treatment isassociated with higher re-fracture risk	1. Larger sample sizes are prone to type 1 error
PubMed	Elvey et al. [33]	Cross-sectional audit	120 patients at a central London hospital	To examine fracture prevention measures following FRAX screening	45% high-risk patients are untreated, and no bone density assessments have been performed	Small sample size; single-centre study
PubMed	Sokhal et al. [34]	Cohort study	Cohort study assessing osteoporotic management in 652 patients treated for polymyalgia rheumatica	To investigate osteoporotic management and fragility fracture risk reduction in patients with polymyalgia rheumatica receiving long-term glucocorticoids	Fewer than 50% of patients received osteoporotic treatment, and only 26% of patients aged 65 and older received anti-osteoporotic medicines.	The study was questionnaire-based, hence fracture and medication use were self-reported, increasing the risk of recall bias.
Web of Science	McEwan et al. [35]	Observational study using semi-structured interviews	30 healthcare professionals in two emergency departments (UK)	To assess barriers to adherence to NICE falls and osteoporosis guidelines	Low compliance with NICE guidance; poor inter-professional communication	Conducted in only two sites; limited generalizability

Critical Appraisal of Individual Sources

Critical appraisal of individual articles was not done as it is not indicated for a scoping review [36].

Synthesis and Analysis of Results

Data were extracted and analysed using the Thematic Content Analysis approach, as described by Naeem et al. [37], including: 1. Data familiarisation; 2. Keyword identification; 3. Code selection; 4. theme development; 5. conceptualisation; 6. Development of our report. The themes were developed using the Socio-Ecological Model (SEM) of health [38]. This model, therefore, helps shape the barriers to optimal osteoporotic treatment into two themes: individual factors and organisational factors, which adequately capture the array of barriers and challenges to prevention of osteoporotic fracture in this scoping review.

Ethical Considerations

The study review did not require obtaining new data from human subjects; therefore, it did not require Brunel Research Ethics Online (BREO) ethics approval. 

Study Limitations

This scoping review was restricted to studies conducted in the UK, potentially excluding relevant findings from international contexts that could offer transferable insights. As a scoping review, it did not involve a critical appraisal of included studies, which may limit the ability to assess the quality and reliability of the evidence. Although a comprehensive search strategy was used, relevant studies may have been missed due to incomplete indexing or database coverage.

Results

A total of 2,124 articles were obtained from the initial database search. The articles included for screening totalled 260 after excluding duplicates and those not relevant to the research question. After screening the titles and abstracts, 39 papers were selected for eligibility review. Finally, 12 articles were included after further screening. See Figure 1 for the PRISMA Flow Chart describing the search strategy and selection process.

Table 4 describes the characteristics of the studies included in the review, with details of the population sample, setting, and study findings. The articles were selected based on eligibility criteria outlined in our search strategy, including only UK studies published between 2014 and 2024, focusing on barriers to optimal osteoporosis management in patients 50 years and older. 

Key findings from the selected literature were analysed using thematic content analysis, with the socioeconomic model used to arrange the findings into relevant themes, as shown in Table 5. Table 6 below provides an overview of the number of supporting articles of the final papers that demonstrate each outcome.

**Table 5:** Socioeconomical model framework [38]

Individual and interpersonal factors	1. Non-adherence to treatment 2. Acceptability of medications 3.Attendance for initial assessments and follow-up appointments 4.Personal characteristics: income level, area of residence, and educational level
Organizational and policy factors	1. Poor coordination of care between primary and secondary centers 2.Poor identification of at-risk patients 3. Delays in initiating treatments 4. Underprescription of osteoporosis medications 5. Poor administration of falls prevention guidelines

**Table 6:** Result analysis for barriers to osteoporotic treatment in the UK

Key findings/evidence	Number of supporting articles	Proportion of selected articles
Underdiagnosis	5	41.7%
Social deprivation	1	8.3%
Poor prescription of prophylactic treatment	3	25%
Poor compliance with falls prevention guidelines	1	8.3%
Treatment delays	4	33.3%
Treatment non-adherence	4	33.3%
Poor care coordination	2	16.7%

Barriers at the Individual Level

At the individual level, four articles found that treatment non-adherence was a barrier to optimal osteoporosis treatment and the prevention of fragility fractures [24-27]. One article linked non-adherence to treatment with cumbersome and too frequent administration of oral medications versus intravenous [27]. One article cited poor attendance for bone density screening among at-risk patients as non-adherence to treatment, posing a significant barrier to reducing the risk of fragility fractures [26].

One article also highlighted social deprivation as a barrier to osteoporotic treatment and a risk factor for fragility fractures [28]. The latter article found that people who lived in low-income areas or with lower educational levels were more likely to have higher rates of falls and poorer access to osteoporotic treatment.

Barriers at the Organisational Level

At the organisational level, five articles reported underdiagnosis or inadequate identification of at-risk patients [29-33]; four identified delays in initiating treatment [29-31, 33], and three papers showed inadequate or poor prescription of anti-osteoporotic medications [24, 32, 34].

In addition, more challenges were identified at the organisational level, including poor coordination of care between primary and secondary centres [30, 31] and poor compliance with falls prevention guidelines [35]. One study found that these barriers were more pronounced among care home residents, as coordination of care between care homes and primary/secondary care centres was significantly impaired, delaying diagnosis, treatment initiation, and treatment monitoring [29].

Discussion

This scoping review demonstrates a persistent, multifactorial treatment gap for osteoporosis in the United Kingdom, despite the availability of evidence-based guidelines and cost-effective interventions. Using the socio-ecological model, the findings highlight how individual behaviours, interpersonal factors, and organisational shortcomings interact to perpetuate underdiagnosis, poor adherence, and delayed treatment initiation. These interconnected barriers help explain why fragility fracture rates remain high and expose important weaknesses in service delivery and equity.

Individual-Level Barriers

Poor medication adherence emerged as a key barrier; however, attributing this solely to patient behaviour oversimplifies the issue. Oral bisphosphonates are associated with complex administration requirements and adverse effects that discourage long-term use. Evidence from the UK SCOOP trial and observational studies shows adherence declines sharply within the first year, reflecting a mismatch between treatment regimens and patients’ capacity for sustained engagement [24].

Socioeconomic deprivation further compounds these challenges. Individuals from lower-income and deprived areas experience reduced access to diagnostic services and treatment, consistent with broader health inequalities in the UK. These barriers intersect with limited health literacy and transport difficulties, creating structural disadvantages that education alone cannot address. Care home residents were consistently identified as a high-risk group, with underdiagnosis, delayed treatment, and poor continuity of care. Fragmented care pathways, cognitive impairment, and low prioritisation of bone health in institutional settings appear to exacerbate these risks.

Organisational and System-Level Barriers

System-level barriers were equally prominent. Underdiagnosis remains common due to limited proactive case-finding in primary care and inconsistent use of fracture risk assessment tools such as FRAX and QFracture. Unequal access to DXA scanning, particularly in rural areas and smaller hospitals, further delays diagnosis and treatment.

Even when osteoporosis is identified, treatment initiation is often delayed due to poor communication between primary and secondary care. Patchy implementation of fracture liaison services (FLS) worsens this fragmentation, despite strong evidence that FLS models reduce refracture rates and improve adherence [29]. Falls-prevention strategies were poorly integrated, particularly in acute care settings, where emergency departments prioritise immediate trauma management over secondary prevention. This represents a missed opportunity for intervention at a critical point in the care pathway.

Implications for Practice, Policy, and Research

These findings call for a shift towards patient-centred care models that simplify treatment regimens, promote shared decision-making, and address social determinants of health. Osteoporosis should be routinely incorporated into long-term condition reviews in primary care, alongside cardiovascular disease and diabetes. Expanding access to DXA scanning, strengthening care coordination, and targeting underserved populations are essential.

Key research gaps remain, including limited evidence on adherence interventions for deprived populations, mobile or community-based DXA services, and the role of integrated electronic health records. Future research should prioritise equity-focused interventions, digital innovations, and longitudinal evaluations of policy impact.

Recommendations

Based on the findings of this review, the following recommendations are proposed for healthcare professionals, policymakers, and public health organisations to reduce the burden of fragility fractures and improve osteoporosis care:

Individual and Interpersonal Level

At an individual or interpersonal level, a key strategy for policymakers and healthcare professionals is to develop nationwide campaigns to educate the public about osteoporosis, its risk factors, and the importance of early diagnosis and treatment. Also, target education programs specifically for older adults, caregivers, and residents in care homes to improve awareness and participation in screening programs.

In addition, healthcare managers need to optimise treatment regimens by encouraging the use of patient-preferred options, such as intravenous bisphosphonates, which have higher adherence rates compared to oral medications. Also, enhance follow-up care by incorporating routine check-ins and patient reminders to support adherence. The shift towards IV bisphosphonates, where appropriate, may reduce the burden of adherence and improve outcomes. However, this requires investment in outpatient infusion services and regular monitoring protocols.

Organisational and Policy Levels

At organisational and policy levels, a key strategy to treating osteoporosis and mitigating osteoporotic fractures is to improve access to diagnostic tools and services across primary and secondary care settings, particularly in underserved regions. Focus diagnostic and treatment services more on high-risk populations, such as postmenopausal women, elderly men, and long-term glucocorticoid users.

Routine osteoporosis screening should be embedded into long-term condition reviews, especially for high-risk groups such as elderly, postmenopausal women, and steroid users. Furthermore, developing integrated fracture liaison services (FLS) to improve continuity of care among primary, secondary, and community health providers is integral to reducing the burden of osteoporosis and osteoporotic fractures. Also, it is essential to establish clear communication protocols and referral pathways to ensure timely diagnosis and initiation of treatment. The expansion of fracture liaison services, with formal integration into electronic health records and referral systems, could mitigate care fragmentation and ensure systematic follow-up. Further, local audits and performance metrics around osteoporosis management (e.g., % of high-risk patients assessed and treated) should be routinely reported and linked to quality incentives in both primary and secondary care.

## Conclusions

This scoping review underscores the critical gaps in osteoporosis management and fragility fracture prevention in the UK, despite the availability of practical evidence-based guidelines and treatments. Fragility fractures, particularly of the hip and vertebrae, impose substantial human and economic burdens, with high mortality and long-term disability risks. The findings highlight that poor adherence to treatment, underdiagnosis, and delays in initiating therapy significantly contribute to the ongoing osteoporosis treatment gap. Factors at both the individual level, such as non-adherence and socioeconomic challenges, and the organisational level, such as care coordination and compliance with guidelines, create barriers to optimal care.

Addressing these challenges demands a comprehensive, multidisciplinary approach that integrates patient education, organisational reform, and policy intervention. Enhancing public and professional awareness, expanding diagnostic capacity, and embedding standardised osteoporosis assessment within long-term condition management can improve early identification and treatment uptake. Strengthening coordination of care through fully resourced fracture liaison services and digital integration across the NHS would help close the treatment gap and ensure continuity between acute and community settings. Ultimately, achieving meaningful progress will require not only clinical excellence but also a societal commitment to prioritising bone health as a public health imperative across the UK.
